# Combined surgery and radiotherapy as curative treatment for tracheal adenoid cystic carcinoma: a case report

**DOI:** 10.1186/s13256-019-1996-9

**Published:** 2019-03-06

**Authors:** Gian Paolo Spinelli, Evelina Miele, Alessandra Anna Prete, Giuseppe Lo Russo, Alessandro Di Marzo, Claudio Di Cristofano, Silverio Tomao

**Affiliations:** 1 UOC of Oncology – ASL Latina – Distretto 1, University of Rome “Sapienza”, Aprilia, LT Italy; 2 Department of Hematology/Oncology and Stem Cell Transplantation, Bambino Gesù Children’s Hospital, IRCCS, Rome, Italy; 3 Radiotherapy Unit, Santa Maria Goretti Hospital, ASL, Latina, Italy; 4 UOC of Pathology, Department of Medical-Surgical Sciences and Bio-Technologies, University of Rome “Sapienza”, Polo Pontino, I.C.O.T., Latina, Italy

**Keywords:** Adenoid cystic carcinoma (ACC), Trachea, Radiotherapy, Surgery

## Abstract

**Background:**

Adenoid cystic carcinoma of the trachea is a rare tumor, characterized by slow growth and low rate of local and distant metastasis. When achievable, complete surgical resection represents the optimal treatment approach, with the highest results in terms of overall survival. Radiation therapy is a reasonable alternative in cases of inoperable disease.

**Case presentation:**

We report a case of an 82-year-old white man affected by primary adenoid cystic carcinoma of the trachea, treated with debulking surgery and radiotherapy on the residual disease.

A three-dimensional conformal radiation therapy was conducted. The total dose amounted to 70 Gy, administered in 35 fractions of 2 Gy. The medium doses given to the esophagus and lungs were 23 Gy and 4.2 Gy respectively. The maximum dose delivered to the spinal cord was 31 Gy with satisfactory results in terms of local control of the disease.

**Conclusion:**

A combined approach of surgical resection followed by radiotherapy on the residual disease provided an excellent result in terms of disease control, quality of life, and overall survival in a patient with locally advanced tracheal adenoid cystic carcinoma.

## Background

Primary tracheal malignant tumors represent 0.1 to 0.4% of all malignant neoplastic diseases and their incidence is approximately 0.1 per 100,000 per year. Malignant tumors of the trachea tend to occur more often in the adult population than in children, but the distribution between the two sexes is almost the same.

Primary adenoid cystic carcinoma (ACC) of the trachea and squamous cell carcinoma (SCC) are the most frequent histological types of tracheal malignant tumors, accounting for approximately two thirds of adult primary tracheal tumors. The other histological classes are very rare and include sarcoma, melanoma, mucoepidermoid carcinoma, lymphoma, and non-squamous bronchogenic carcinoma.

ACC seems not to be related to tobacco smoking, and its prognosis is generally better than SCC, which progresses faster and frequently metastasizes. In contrast, only 10% of patients affected by ACC present with locoregional or distant metastases. The main growth patterns of tracheal ACC documented in histological samples are tubular, solid, and cribriform, the latter is the most frequently observed.

Local recurrences are not uncommon in ACC, but they are frequently late. Radical surgery is related to the best outcomes in overall survival; in unresectable tumors, radiotherapy ensures satisfactory results in terms of local control of the disease, and is adequate to treat residual disease after surgery [1, 2].

Here we report a case of such rare pathology that we managed it with a combinatorial strategy of surgery and radiotherapy which allowed adequate disease control.

## Case presentation

We report a case of an 82-year-old white man, who never smoked tobacco or consumed alcohol, who presented with a 3-month history of tracheitis and dysphonia. His past medical history was characterized by multiple myeloma, Gilbert syndrome, chronic obstructive pulmonary disease (COPD) treated with bronchodilators, cardiac arrhythmia treated with amiodarone, and arterial hypertension treated with angiotensin-converting enzyme (ACE) inhibitors.

On arrival, his physical signs were as follows: oriented, collaborating, and autonomous walking; a neurological examination showed no abnormalities; blood pressure 130/85 mmHg and pulse 80 beats/minute; no fever; and regular bowel function and diuresis. Routine laboratory tests were performed, including complete blood count, renal and liver function tests, and electrolytes. All the results of the laboratory tests were almost within normal range.

A frontal and lateral chest radiograph was performed as first imaging procedure: it showed prominent pulmonary hila and a reduction of vascular marking, but no nodular lesions or neoformations were documented. Therefore he underwent a total body computed tomography (CT) scan without contrast, due to the multiple myeloma, which revealed the presence of massive hyperdense solid tissue in the mid-proximal trachea, protruding into the lumen. This neoformation determined compression and narrowing at the level of the anterior-lateral wall of the right portion of his esophagus.

**Fig. 1:**
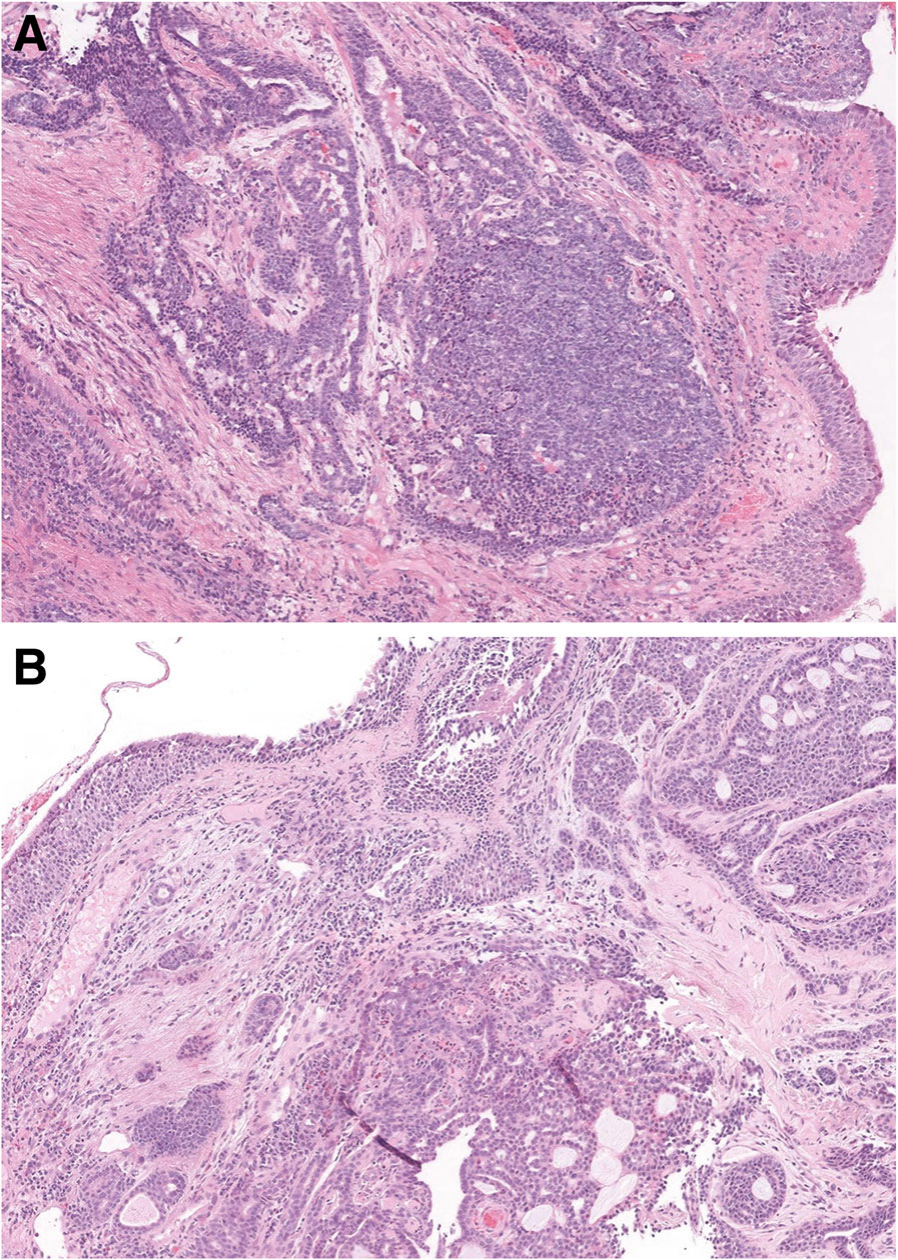
**a**, **b** Neoplastic proliferation composed of small cells with basophilic nucleus, inconspicuous nucleolus and scant cytoplasm arranged in nodules with cribriform pattern, cords, and tubules of varying size, containing eosinophilic material

Thus, he underwent a bronchoscopy that confirmed tracheal lumen narrowing between the first and fifth tracheal ring. A biopsy specimen of the lesion revealed a salivary gland-type neoplasm, showing a moderate degree of aggressiveness, with the characteristics of ACC (Fig. 1a, b).

Due to the rarity of the neoplasm, with less than two cases in 1 year in our institute, a review of the literature was made. A multidisciplinary team of oncologists, radiologists, radiotherapists, and surgeons decided to treat our patient with an endotracheal debulking surgical excision of the lesion followed by radiotherapy.

**Fig. 2:**
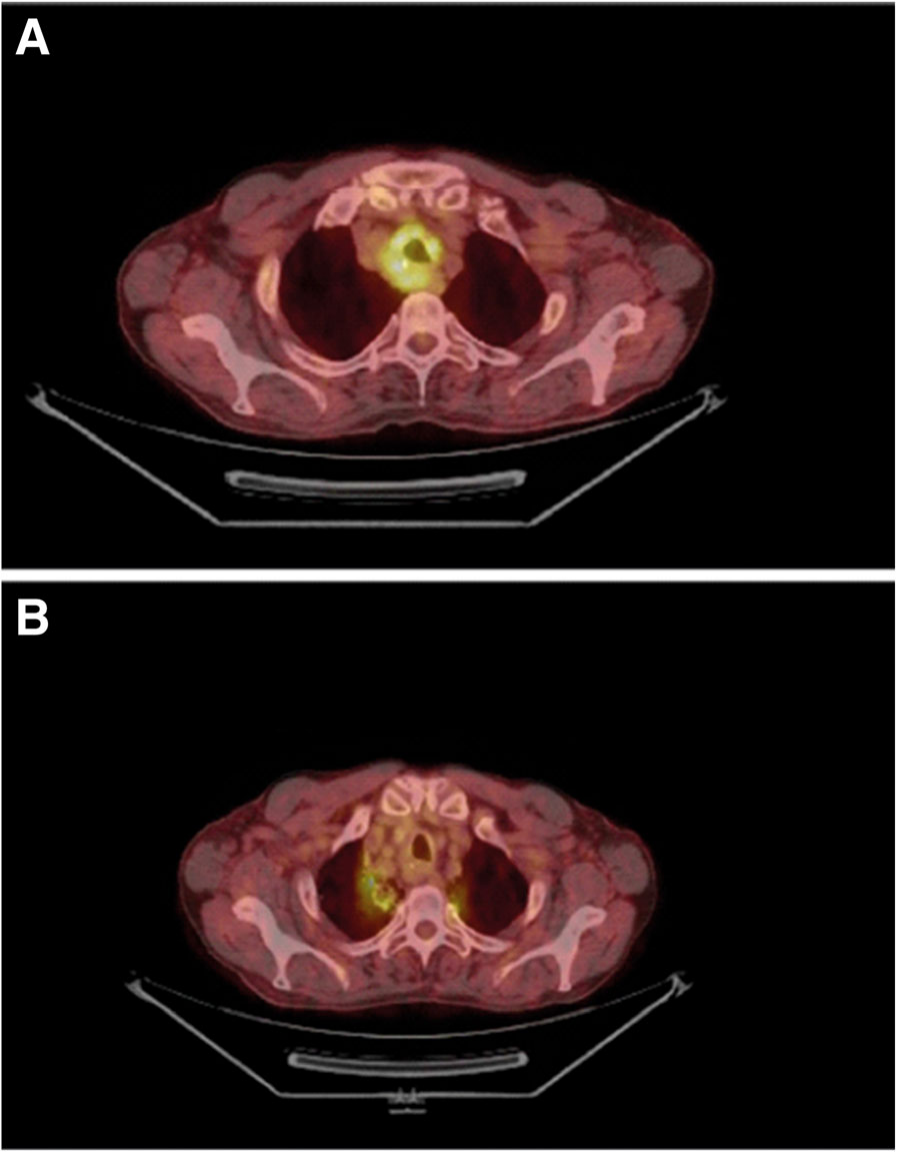
**a**, **b** Positron emission tomography-computed tomography before (**a**) and after (**b**) radiotherapy. The increased uptake of ^18^F-fluorodeoxyglucose at the level of trachea is not visible after the treatment. The increased uptake of ^18^F-fluorodeoxyglucose in correspondence with the superior pulmonary lobes refer to the local inflammation caused by radiotherapy

Three weeks after the surgery, a positron emission tomography (PET)/CT scan was performed: a residual solid tissue with a maximum diameter of 46 mm was evident in the middle mediastinum, infiltrating the upper middle third of his trachea and showing strong ^18^F-fluorodeoxyglucose (18-FDG) uptake (Fig. 2a). A three-dimensional conformal radiation therapy (3D-CRT) was conducted. The target volume was determined by CT. Lungs, heart, left coronary artery, and spinal cord were identified as organs at risk of accidental irradiation. The radiotherapy was delivered with linear accelerator of photons. The total dose amounted to 70 Gy, administered in 35 fractions of 2 Gy. The medium doses given to his esophagus and lungs were 23 Gy and 4.2 Gy respectively. The maximum dose delivered to his spinal cord was 31 Gy.

After 1 year of follow-up, no early or late toxicities related to the radiotherapy were observed: there was no dysphagia or weight loss.

PET-CT scans performed after 6 and 12 months of follow-up showed a complete response to the radiotherapy, with only a focal increased uptake at the level of superior pulmonary lobes, which referred to post-radiotherapy inflammation (Fig. 2b).

## Discussion

ACC of the trachea is characterized by slow growth and low rate of local and distant metastasis.

We report the case of a patient with the rare condition of tracheal ACC that was successfully treated with a combination of surgery and radiotherapy.

CT and magnetic resonance proved to be the most appropriate imaging techniques for the characterization of the primary tumor and to document lymph nodal or distant metastases. A PET-CT scan is also useful for staging and has been used to direct intensity-modulated radiation therapy (IMRT) treatment.

A better prognosis is mainly related to the feasibility of complete resection and to the absence of metastasis. However, symptoms such as wheezing, tracheitis, hemoptysis, and stridency are often unspecific and late. Lymph nodal involvement has not been associated with a worse prognosis [3, 4].

The overall survival at 5 years of tracheal ACC is approximately 30% with a reduction to 18% in cases of extratracheal extension. In cases of intraluminal disease, all the studies shows a 5-year overall survival rate of 50% [5].

The optimal treatment approach for ACC consists of radical surgical resection with negative margins achievement and reconstruction [2]. Surgical techniques depend on the anatomic region in which the tumor develops. In cases of tumors involving the proximal trachea, concomitant laryngectomy can be required, and a cervical or mediastinal-end tracheostomy is necessary. In cases of a standard resection, an end-to-end anastomosis is usually performed. Even when a radical surgical intervention is theoretically possible, some practical limitations can present [2].

When complete resection cannot be achieved, postoperative radiotherapy seems to have a statistically significant impact on survival [6–8]. ACC of the trachea, in fact, proved to be radiosensitive. Our patient experienced excellent disease control, with a good quality of life, and prolonged overall survival by combining surgery and radiotherapy.

Although there is not an accepted standard, usually the dosage of postoperative radiotherapy is 60 Gy (2 Gy per fraction, five fractions per week, for a total of 6 weeks). Endotracheal brachytherapy can be used to give a boost on the primary tumor or to treat advanced stages with palliative intent [1].

In cases of inoperable disease, radiotherapy alone can reach a sufficient local control of the disease, but it does not give results comparable to those of surgery. Palliation can be obtained with several procedures, such as electrocoagulation, cryotherapy, photodynamic therapy, or with stents, above all in cases of severe airway obstruction.

Many studies are available in the scientific literature about the employment of radiotherapy in ACC (Table 1). Maziak *et al.* [6] published a retrospective study in which they analyzed 38 patients affected by ACC of the upper airways and treated by surgery and/or adjuvant radiotherapy. No statistically significant difference between the group of patients treated with adjuvant radiotherapy and the group treated with resection only was found. However, the authors assumed adjuvant radiotherapy to be beneficial for local control and recurrence prevention. This observation is in agreement with the conclusions drawn by Grillo and Mathisen [4].

**Table 1:** Studies regarding radiotherapy employment in the treatment of tracheal tumors

Author, year of publication	Type of study	Disease	Treatment	Outcome
Maziak *et al*., 1996 [6]	Retrospective study	ACC of the upper airways	− 38 patients underwent resection: 25 received adjuvant radiotherapy (various dosages); 7 did not receive adjuvant therapy − 6 inoperable patients treated with primary radiotherapy (50 to 75 Gy)	– Mean survival with adjuvant radiotherapy: 88 ± 76 months – Mean survival with resection only: 131 ± 131 months – Mean survival with primary radiotherapy alone: 74 ± 64 months
Kanematsu *et al*., 2002 [7]	Retrospective study	Primary ACC of the lung	− 5 of 6 patients underwent resection and received adjuvant RT (50–61.2 Gy) − 5 patients with unresectable tumor underwent primary RT (50 to 70 Gy)	At a 5-year follow-up: – survival rate in patients treated with adjuvant RT: 91% – survival rate in patients treated with primary RT: 40%
Regnard *et al*., 1996 [3]	Multicenter retrospective study	Primary tracheal tumors, including ACC	26 of 60 patients underwent resection and received adjuvant therapy	Radiotherapy showed beneficial effects on survival in cases of incomplete resection only
Grillo and Mathisen, 1990 [4]	Series of cases	Primary tracheal tumors, including ACC	60 of 80 patients affected by ACC underwent surgical resection, and nearly all of them received postoperative RT; 12 patients treated with RT only (9 of which died of carcinoma)	Resection associated to RT gives 2 to 4 years survival

*ACC* adenoid cystic carcinoma, *RT* radiotherapy

Kanematsu *et al.* [7] in a retrospective study reported a long-term survival with 5-year follow-up in patients affected by primary ACC of the lung treated with either adjuvant radiotherapy or palliative radiotherapy in cases of unresectable cancer. After a 5-year follow-up the survival rate of patients treated with surgery and adjuvant radiotherapy was 91%, while in the unresected group the survival rate amounted to 40%.

Le Pechoux *et al.* [8] proved a survival rate of 65–80% at 5-year follow-up in patients affected by ACC of the trachea treated with surgery and adjuvant radiotherapy and of 12–27% in patients with unresectable tumors treated with radiotherapy only.

Regnard *et al*. [3] conducted a retrospective multicenter study in which 208 patients affected by primary tracheal carcinomas were enrolled. Of these patients, 65 were affected by ACC of the trachea. This study showed a beneficial effect of adjuvant radiotherapy on survival only for patients with incomplete resection of the tumor.

Bonner Millar *et al.* [9] compared two patients affected by tracheal ACC and treated successfully with definitive radiotherapy: one of them was treated with conventional photons; the other one was treated with a combination of photons and protons. The former had no evidence of disease at a 5-year follow-up; the latter had no evidence of disease at 11 months of follow-up.

Bittner *et al*. [10] published a retrospective study in which 20 patients affected by ACC of the trachea were treated with fast neutron radiotherapy, six of these patients received boost by endobronchial brachytherapy. At a 46-month follow-up, no statistically significant difference in overall survival and of locoregional control rate was found between the two groups.

Radiotherapy should always be performed in order to cover the entire tumor bed and to spare the surrounding normal tissues. In cases of treatment of tracheal tumors, preventing near structures (such as esophagus, lungs, and spinal cord) from accidental irradiation is particularly important to limit side effects and late complications. Alongi *et al.* [1] in a case report described the employment of helical tomotherapy as adjuvant treatment for a patient affected by tracheal ACC. Helical tomotherapy is a technique that performs IMRT with a helical delivery pattern. This technique allows a more precise irradiation of the tumor bed, minimizing accidental irradiation of normal structures. There is no evidence about the usefulness of chemotherapy in the treatment of tracheal ACC.

## Conclusions

In conclusion, due to the rarity of the disease and thus the lack of randomized clinical trials, there is not yet a standardized therapeutic approach to tracheal ACC. In our experience, considering the slow tumor growth and despite our patient’s short follow-up, the combinatorial strategy of a debulking surgery followed by postoperative radiotherapy successfully achieved adequate disease control, suggesting it would be considered in the treatment option of locally advanced ACC of the trachea.
